# Catalytic Antioxidants in the Kidney

**DOI:** 10.3390/antiox10010130

**Published:** 2021-01-18

**Authors:** Yu Ah Hong, Cheol Whee Park

**Affiliations:** 1 Department of Internal Medicine, College of Medicine, The Catholic University of Korea, Seoul 06591, Korea; amorfati@catholic.ac.kr; 2 Institute for Aging and Metabolic Diseases, College of Medicine, The Catholic University of Korea, Seoul 06591, Korea

**Keywords:** catalase, glutathione peroxidase, superoxide dismutase, catalytic antioxidants, kidney

## Abstract

Reactive oxygen species and reactive nitrogen species are highly implicated in kidney injuries that include acute kidney injury, chronic kidney disease, hypertensive nephropathy, and diabetic nephropathy. Therefore, antioxidant agents are promising therapeutic strategies for kidney diseases. Catalytic antioxidants are defined as small molecular mimics of antioxidant enzymes, such as superoxide dismutase, catalase, and glutathione peroxidase, and some of them function as potent detoxifiers of lipid peroxides and peroxynitrite. Several catalytic antioxidants have been demonstrated to be effective in a variety of in vitro and in vivo disease models that are associated with oxidative stress, including kidney diseases. This review summarizes the evidence for the role of antioxidant enzymes in kidney diseases, the classifications of catalytic antioxidants, and their current applications to kidney diseases.

## 1. Introduction

**Figure 1:**
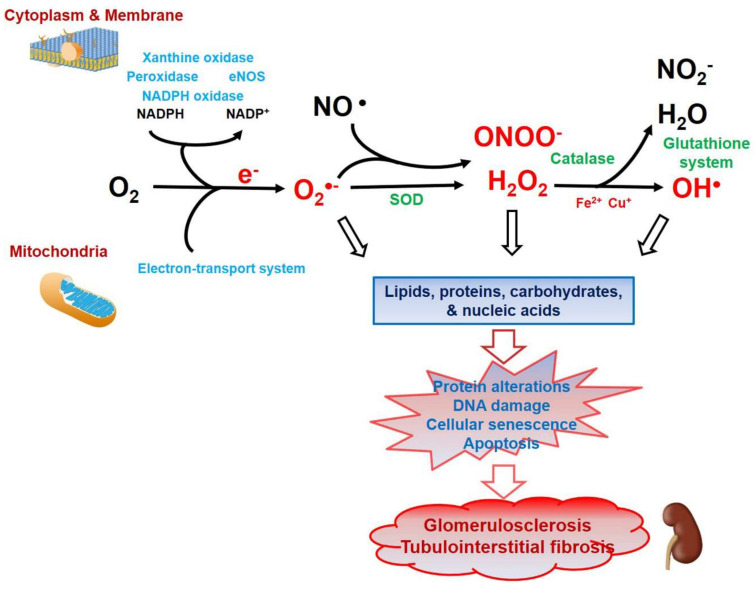
Schematic overview of endogenous sources of oxidative stress and antioxidative reactions in renal damage. Exogenous (environmental factors such as air and water pollution, smoking, drugs, and radiation) and endogenous (normal metabolic processes in living organisms) sources of oxidative stress produce reactive oxygen species (ROS). Endogenously, ROS are generated as products of biochemical reactions in the mitochondria (electron-transport system; ETS), plasma membrane, cytoplasm (including peroxisomes and lysozymes), and the membrane of the endoplasmic reticulum. The mitochondrial ETS, adenine dinucleotide phosphate (NADPH) oxidase, xanthine oxidase, myeloperoxidase, and endothelial nitric oxide synthase (eNOS) are the main sources of cellular ROS formation. An important reaction in free radical formation is the Fenton and Fenton-like reactions to produce ROS in which Fe^2+^ and Cu^+^ react with H_2_O_2_ to form OH^•^, respectively. To protect and repair the molecular injury caused by ROS, cells use a defense system composed of enzymatic antioxidants, including superoxide dismutase (SOD), catalase, and peroxidase, and nonenzymatic antioxidants made by the glutathione system. The main site of O_2_^•−^ generation is the inner mitochondrial membrane during ETS-processes. The decomposition of H_2_O_2_ into water and oxygen is done by SOD, the glutathione system, and catalase, in that order. Excess ROS causes lipid peroxidation, nitro-oxidation, glyco-oxidation, and oxidative DNA damage, which can together cause protein alterations, DNA damage, cellular senescence, and apoptosis. All of those changes eventually lead to glomerulosclerosis and tubulointerstitial fibrosis.

Oxidative stress describes an imbalance between the formation of reactive species and the defense of antioxidants that occurs to a disturbance in redox signaling or molecular damage [1]. Reactive oxygen species (ROS) and reactive nitrogen species (RNS) are generated as toxic byproducts of the oxygen metabolism that is essential for living organisms. These free radicals consist of superoxide (O_2_^•−^), hydrogen peroxide (H_2_O_2_), nitric oxide (NO^•^), hydroxyl radicals (OH^•^), peroxynitrite (ONOO^−^), and lipid peroxyl radicals (LOO^•^) [1]. During respiration, cellular O_2_^•−^ is produced endogenously in the mitochondria, and ROS are generated by complexes in the electron transport chain and partially reduced metabolites of molecular oxygen formed in biological systems [2]. Excessive ROS production develops through the activation of specific oxidases, including nicotinamide adenine dinucleotide phosphate (NADPH) oxidase (NOX), xanthine oxidase, uncoupled nitric oxide synthase (NOS), and arachidonic acid-metabolizing enzymes [3]. ROS can induce the damage of cellular proteins, lipids, carbohydrates, and DNA, finally leading to cellular dysfunction. Therefore, they are being explored since early times as important modulating agents in numerous cellular signaling pathways (Figure 1) [4]. Antioxidant defense mechanisms are complicated and compartmentalized, enabling the independent regulation of cytoplasmic, mitochondrial, and nuclear levels of ROS [5]. ROS levels are regulated in living systems by numerous antioxidant enzymes, including superoxide dismutase (SOD), catalase (CAT), glutathione peroxidase (GPx), peroxiredoxin (Prx), thioredoxin (Trx), and cytochrome c oxidase [6,7].

Oxidative stress is implicated in the pathogenesis of various kidney diseases, including acute kidney injury (AKI), chronic kidney disease (CKD), hypertensive nephropathy, and diabetic nephropathy [8,9,10,11]. Therefore, antioxidant agents are promising therapeutic strategies for kidney disease. Catalytic antioxidants are small, molecular mimics of antioxidant enzymes such as SOD, CAT, and GPx, and some of them act as detoxifiers of lipid peroxides and ONOO^−^ [12]. Since these compounds are catalytic and not simply free-radical scavengers, they display more potent antioxidant activity than other dietary supplements [12]. This article summarizes the evidence for the role of antioxidant enzymes in kidney disease, the classifications of catalytic antioxidants, and their current applications to kidney diseases.

## 2. Antioxidant Enzymes and Kidney Disease

Cells have crucial antioxidant defense mechanisms to protect themselves against toxic injury by free-radicals. Antioxidants can have endogenous or exogenous origins, with endogenous synthesis producing enzymes and small molecules or diet providing important exogenous defenses. Based on their activity, antioxidants can be divided into enzymatic and non-enzymatic. The primary enzymatic antioxidants are SOD, CAT, and GPx. Endogenous non-enzymatic antioxidants include L-arginine, lipoic acid, coenzyme Q10, melatonin, albumin, and uric acid [13]. Exogenous non-enzymatic antioxidants include nutrients such as ascorbic acid (vitamin C), α-tocopherol (vitamin E), phenolic antioxidants, lecithin oil, and drugs such as acetylcysteine [14]. There are also several antioxidant systems in the kidney to protect renal tissue and related cells from oxidative stress.

### 2.1. Superoxide Dismutase and Kidney Disease

Superoxide radical anions are a potentially harmful species produced from the one-electron reduction of molecular oxygen during respiration. SODs are key antioxidant enzyme systems, and most organisms that live in the presence of oxygen express at least one SOD. The coordinated metals at the active site can be used to classify SODs: copper-zinc SOD (Cu/Zn-SOD), manganese SOD (Mn-SOD), iron SOD (Fe-SOD), and nickel SOD (Ni-SOD) [4]. As a group of metalloenzymes that catalyze dismutation reactions to detoxify ROS [12], SODs catalyze the dismutation of two O_2_^•−^ to yield H_2_O_2_ and molecular O_2_, which is decomposed into water and oxygen through CAT [15].

2O_2_^•−^ + 2H_3_O^+^ → O_2_ + H_2_O_2_ + 2H_2_O (1)

SODs are also classified into three major isoforms based on their localization in subcellular compartments: SOD1 (Cu/Zn-SOD), SOD2 (Mn-SOD), and SOD3 (Extracellular SOD, EC-SOD), all of which are normally presented in the kidney. SOD1 constitutively exists in the cytosol and intermembrane space on mitochondria, and SOD2 is found in the mitochondria of eukaryotic cells. SOD3 is a Cu/Zn-SOD that is secreted into the extracellular space [16]. Of those three SODs, SOD1 is abundant in most tissues and accounts for 60–80% of SOD activity and about 30% of SOD activity in the renal vasculature in the kidneys [17]. SOD2 is also expressed in most tissue cells, e.g., stomach, lung, skeletal muscle, spleen, heart, liver, kidney, and brain [18]. SOD3 is highly expressed in the blood vessels, kidneys, lungs, and heart [4]. Although SOD1 accounts for the highest proportion of SOD activity in the kidney, SOD2 deficiency has been associated with more severe pathological changes than SOD1 deficiency [19], because ROS and RNS are mainly formed in the mitochondria [11].

All three of the SOD isoforms play a crucial role in the deterioration and alleviation of various kidney diseases. Several experimental studies have provided evidence that deleting or overexpressing SODs through genetic manipulation or medication changes oxidative stress and the disease severity of AKI or CKD. The depletion of SOD1 causes a significant increase in nuclear factor κ light chain enhancer of activated B cells (NF-κB)-mediated signal transduction and oxidative DNA damage in the kidneys [20,21]. Indeed, SOD1 knockout mice had severely decreased renal function after renal ischemia/reperfusion (I/R) injury [22], and treatment with recombinant human SOD1 significantly decreased ROS and improved renal function by decreasing tissue levels of tumor necrosis factor (TNF)-α and interleukin (IL)-1 in renal I/R injury [23]. SOD1 deficiency augmented salt sensitive–hypertension and tubulointerstitial fibrosis in unilateral ureteral obstruction (UUO) mice, whereas SOD1 overexpression using transgenic mice or chronic tempol treatment abolished those findings in UUO mice [24]. SOD1 also regulates renal microvascular remodeling, arteriolar responsiveness, and sensitivity to angiotensin II (Ang II). SOD1 knockout mice displayed increased blood pressure and decreased afferent arteriole diameter during Ang II infusion, and those changes were mitigated in SOD1-transgenic mice [25]. In diabetic nephropathy, advanced glycation end products (AGEs) augmented oxidative stress via the ROS production by NOX in the mitochondria, and the interaction between AGEs and the receptor of AGEs (RAGE) enhanced the initiation of related signal transduction [26]. Antioxidant enzymes, such as SOD and CAT inhibit AGE-mediated ROS production. SOD1-transgenic db/db mice and streptozotocin (STZ)-treated SOD1-transgenic mice exhibited reduced albuminuria, transforming growth factor (TGF)-β1, and collagen IV expression, along with mesangial matrix expansion and decreased oxidative stress markers compared with control diabetic mice [27,28].

SOD2 dysfunction has been reported to aggravate renal dysfunction, tubulointerstitial fibrosis, inflammation, and apoptosis in the kidney [29]. Parajuli et al. found that kidney-specific SOD2-deficient mice had lighter and smaller kidneys than wild type mice and enhanced oxidative stress and tubular injury, including the dilation of distal tubules, protein cast formation, and epithelial cell swelling in distal tubules [30]. In renal I/R injury, SOD2 knockout mice exhibited lower expression of SOD2 in the distal nephrons and exacerbated renal function compared with control mice [31]. Pretreatment with recombinant SOD2 significantly increased SOD activity and ameliorated renal function declines and tubular necrosis in a rat model of radiocontrast-induced AKI [32]. Furthermore, a high salt diet in SOD2-deficient mice caused a significant increase in arterial pressure and urinary albumin excretion through the upregulation of NOX and the activation of NF-κB [33]. Another study also showed that SOD2 deficiency aggravated renal interstitial inflammation and accelerated glomerulosclerosis, tubulointerstitial damage, and salt-sensitive hypertension, especially in aged mice [34]. The mechanism of impaired microvascular function proposed by those authors was that SOD2 deficiency increased O_2_^•−^ levels and impaired the flow and agonist-induced vasodilation of isolated mesenteric arteries [35].

Excess mitochondrial O_2_^•−^ production and related mitochondrial dysfunction have been associated with the pathogenesis of diabetic nephropathy [36,37]. Several experiments have reported reductions in SOD2 activity in animal models of type 1 and type 2 diabetic nephropathy [38,39,40]. In contrast, other studies have reported no significant differences in SOD2 expression between diabetic and control mice [41,42]. Dugan et al. showed that SOD2-deficient mice with diabetes had increased renal ROS, but they found no evidence of an increase in albuminuria or mesangial matrix expansion [43]. Therefore, the role of SOD2 in diabetic nephropathy is controversial, and additional research is needed to determine the mechanisms of SOD2 activity in diabetic nephropathy.

As with SOD1 and SOD2, several studies have used SOD3 knockout animal models to demonstrate the role of SOD3 in protecting against or accelerating kidney damage in response to oxidative stress. Ang II treatment after renal artery clipping in SOD3 knockout mice developed higher blood pressure and induced endothelial dysfunction, and recombinant SOD3 treatment selectively decreased blood pressure in hypertensive SOD3 knockout mice [44]. Another study reported that SOD3 localizes predominantly in the proximal tubules and colocalizes with erythropoietin (EPO). Compared with the control animals, hypoxia-exposed SOD3 knockout mice showed smaller increases in their EPO levels and lesser accumulations of nuclear translocated hypoxia-inducible factor (HIF)-1α by the activation of NOX in the kidneys [45]. In line with that finding, the deletion of SOD3 blunted renal blood flow recovery after renal ischemia and significantly increased tubular necrosis and tubular cast formation after reperfusion [46]. SOD3 knockout mice also had increased proteinuria and renal fibrosis and podocyte injury after adriamycin treatment, an experimental model of focal segmental glomerulosclerosis (FSGS), and that finding was associated with an upregulation of NOX2 and β-catenin signaling [47]. Therefore, SOD3 plays a crucial role in renal protection against diverse kidney diseases.

To assess the role of SOD isoforms in diabetic nephropathy, Fijuta et al. evaluated SOD activity and SOD isoform expression in the kidneys of diabetic mouse models and found the downregulation of SOD1 and SOD3, but not SOD2, in diabetic kidneys [42]. The same group reported using SOD1- and SOD3-knockout diabetic mice to confirm the distinct role of SOD isoforms in diabetic nephropathy [48]. They suggested that SOD1 deficiency, but not SOD3 deficiency, increases renal O_2_^•−^ and causes overt renal injury in C57BL/6-Akita diabetic mice and that SOD1 plays a more prominent role than SOD3 in the pathogenesis of diabetic nephropathy. However, recent studies have reported that SOD3 has an independent role in protection against diabetic nephropathy [49,50]. Our study demonstrated that the expression of SOD3 was significantly increased in the glomerulus and tubular area of db/db mice after recombinant human SOD3 supplements [50]. Recombinant human SOD3 supplements ameliorated diabetic nephropathy by inhibiting ROS and the phosphorylation of extracellular signal-regulated kinase (ERK)1/2 or the activation of intrarenal 5′-AMP-activated protein kinase–peroxisome proliferator-activated receptor γ coactivator (PGC)-1α–nuclear factor erythroid-2-related factor (Nrf)2 signaling in animal models of type 1 and type 2 diabetic nephropathy [49,50]. Therefore, further experiments are needed to clarify the independent role of SOD3 in protecting against diabetic nephropathy.

### 2.2. Catalase and Kidney Disease

CAT is a 240-kDa homotetrameric heme-containing protein located predominantly in the peroxisome and abundantly present in the liver, lungs, and kidneys [51]. In the kidney, CAT is largely distributed in the cytoplasm of proximal tubules of the juxtamedullary cortex but is less expressed in the proximal tubules of the superficial cortex. On the other hand, CAT is not present in the glomeruli, distal tubules, loop of Henle, or collecting ducts [52]. CAT deficiency results in the overexpression of mitochondrial ROS and functional mitochondrial impairment [53]. CAT reduces the H_2_O_2_ generated by SOD into oxygen and water. Although CAT is highly efficient at reducing H_2_O_2_, its role in modulating H_2_O_2_ might not be central because it is mainly localized in the peroxisome.

2H_2_O_2_ → 2H_2_O + O_2_ (2)

CAT deficiency has been reported to increase tubulointerstitial fibrosis and the lipid peroxidation products of tubulointerstitial lesions in UUO mice [54]. Kobayashi et al. confirmed that CAT decreased renal function and accelerated progressive renal fibrosis through the upregulation of the epithelial to mesenchymal transition in the remnant kidneys of acatalasemic mice subjected to 5/6 nephrectomy [55]. In addition, adriamycin treatment in acatalasemic mice produced severe albuminuria, accelerated glomerulosclerosis and tubulointerstitial fibrosis, and enhanced lipid peroxide accumulation compared with wild-type mice [56].

In diabetic nephropathy, proximal tubule-specific overexpression of CAT inhibited renal ROS production and tubulointerstitial fibrosis and attenuated angiotensinogen, p53, and proapoptotic Bcl-2 associated X-protein (BAX) gene expression in STZ-treated diabetic mice and db/db mice [57,58]. Consistent with those studies, CAT overexpression in Akita mice significantly decreased systolic blood pressure by regulating the intrarenal renin-angiotensin system (RAS), which enhanced angiotensin-converting enzyme (ACE)2 and suppressed ACE and angiotensinogen expression [59], or by activating the nuclear factor erythroid 2–related factor 2 (Nrf2)-heme oxygenase (HO)-1 signaling pathway [60]. Godin et al. confirmed the association between CAT and intrarenal RAS actions in the development of hypertension and renal injury using proximal tubule–specific CAT and/or angiotensinogen transgenic mice [61]. Another researcher also reported that CAT deficiency accelerated diabetic nephropathy by impairing peroxisomal/mitochondrial biogenesis and fatty acid oxidation [53]. Therefore, endogenous CAT plays an important role in protecting against diabetic nephropathy by decreasing oxidative stress through the regulation of intrarenal RAS and peroxisomal metabolism.

### 2.3. Glutathione Peroxidase and Kidney Diseases

GPx, another H_2_O_2_ scavenger, converts peroxides and OH^•^ into nontoxic substances by oxidizing reduced glutathione (GSH) into glutathione disulfide (GSSG), which is then reduced back to GSH by glutathione reductase using NADPH [62,63]. GPx cooperates with CAT to decompose H_2_O_2_ to H_2_O and oxidized glutathione, which is then reduced by glutathione reductase. GPx requires GSH as a hydrogen donor to decompose H_2_O_2_ into water and oxygen and selenium (Se) as a cofactor to participate in the reaction with peroxides [64].

GPxs are tetrameric proteins in which each monomer includes one atom of Se at the catalytic site. Each monomer contains a selenocysteine, which the sulfur in the cysteine has been replaced by selenium (R-SeH). Throughout the catalytic cycle, a selenol (protein-Se^−^) reacts with peroxide (H_2_O_2_ or lipid hydroperoxide, LOOH) to produce selenenic acid (protein-SeOH). Selenenic acid regenerates selenol by two GSH, and GSH are finally oxidized into a GSSG and LOOH. LOOH is reduced to its corresponding lipid alcohol (LOH) [65].

H_2_O_2_ + 2GSH → GSSG + 2H_2_O
LOOH + 2GSH → GSSG + H_2_O + LOH (3)

To date, eight different GPxs have been found in mammals; however, only five isoforms contain the selenocysteine needed to catalyze the reduction of H_2_O_2_ and LOOHs using GSH as a reducing cofactor (GPx 1–4 and 6) [66]. In the kidney, substantial amounts of GPx have been found in the proximal and distal tubules and smooth muscle cells of the renal arteries [67]. Among the GPx isoforms, GPx1 and GPx4 expression is mainly detected in podocytes and mesangial cells [68]; GPx3 is produced in the basement membranes of the renal cortical proximal and distal convoluted tubules [69]; and GPx2 and GPx5 have not been detected in the kidney. As the first to be identified, GPx1 is highly expressed throughout the human body, and its role in the reduction of oxidative stress has been widely demonstrated [67]. GPx1 predominantly exists in normal kidneys, accounting for 96% of kidney GPx activity [70,71]. Esposito et al. demonstrated that GPx1 is substantially expressed in the mitochondria of the kidney cortex, and GPx1 deficiency reduced body weight and exacerbated an endogenous, age-dependent decline in overall cellular function [72]. Therefore, regulation of kidney GPx1 was postulated to play a principal role in protecting kidneys from oxidative stress [71].

Several previous studies have been evaluated the renoprotective role of GPx1 against kidney diseases. Genetic inhibition of GPx1 aggravated cocaine-induced AKI by activating the angiotensin II type-1 receptor (AT1R) through the inhibition of phosphoinositide 3-kinase (PI3K)-Akt signaling [73]. In addition, GPx1 overexpression improved glomerulosclerosis by attenuating oxidative stress and mitochondrial ROS in aged mice [74]. In diabetic nephropathy, Chiu et al. reported that plasma and urine GPx levels were substantially lower in patients with diabetic glomerulosclerosis than in those without glomerulosclerosis and that glomerular GPx expression was lower in diabetic rats than in the normal control rats [75]. However, GPx1-deficient diabetic mice showed levels of oxidative injury, glomerular damage, and renal fibrosis similar to those found in the control diabetic mice, and GPx1 deficiency was not endogenously compensated by the increases of CAT or other GPx isoforms in the early stage of diabetic kidney disease [71]. Enhanced GPx activity and GPx carboxylation did not accompany a concomitant increase in GPx expression in the kidneys of young diabetic mice, and GPx1 and GPx4 expression and activity in the kidney did not differ between aged diabetic and non-diabetic mice [68]. In contrast, Chew et al. demonstrated that in diabetic ApoE/GPx1 double knockout mice, GPx1 deficiency increased albuminuria, which is associated with increased mesangial matrix expansion and the upregulation of inflammatory and fibrotic mediators [76]. Therefore, the renoprotective role of GPx1 against diabetic kidney disease remains uncertain.

GPx3 is an extracellular antioxidant selenoprotein that is also called *plasma GPx* [77]. GPx3 is mainly synthesized in the basolateral compartment of the kidney and binds to the basement membrane of renal cortical epithelial cells [69]. GPx3 also binds to basement membranes of extra-renal epithelial cells in the gastrointestinal tract, lung, and epididymis though the bloodstream [78]. These findings suggest that GPx3 deficiency due to kidney injury may affect the remote organ. Indeed, GPx3 deficiency significantly decreased survival rates and promoted left ventricular dysfunction due to a ROS accumulation that exacerbated inflammatory signaling and platelet activation in a surgery-induced CKD model [79]. Therefore, GPx3 may play an important role in the crosstalk between kidney and other organs.

Recently, ferroptosis, an iron-dependent programmed cell death characterized by accumulating lipid hydroperoxides to lethal levels, has been reported to be involved in the pathophysiology of various renal diseases [80,81,82]. GPx4 is the primary enzyme that prevents ferroptosis, and a GPx4 inhibitor induced ferroptotic cell death by binding and inactivating GPx4 [83]. GPx4 deficiency also aggravated AKI through an increase of intracellular LOOH and the promotion of ferroptotic cell death; liproxstatin-1 prevented kidney injury associated with GPx4 depletion [84]. A recent study showed that diabetic mice had significantly increased levels of acyl-CoA synthetase long-chain family member 4 (ACSL4) and decreased GPx4, and those findings suggest that ferroptosis was involved in the pathogenesis of diabetic nephropathy [85]. To date, no association has been elucidated between GPx2 and GPx5 and renal disease.

## 3. Catalytic Antioxidants

Excessive ROS produces oxidative damage to cellular structures through an imbalance in the oxidant–antioxidant status, and therefore, antioxidants can be used therapeutically to recover the balance between ROS generation and removal [86]. Several exogenous, native antioxidants have proved unsuccessful as therapeutic strategies because of their short half-life, low cell permeability due to their large size, antigenicity, and high-manufacturing costs [12,87]. Catalytic antioxidants have caught the attention of experts for the treatment of diseases associated with ROS. Several catalytic antioxidants have been designed and developed based on the structures of the active sites of native antioxidative enzymes. Therefore, they have been documented to exhibit SOD activity, ONOO^−^-reduction activity, CAT activity, and GPx activity.

Catalytic antioxidants can be classified as independent catalytic antioxidants (ICAs) and cofactor-dependent catalytic antioxidants (DCAs) by how they perform their catalytic action [88]. ICAs decompose ROS/RNS without the need for any additional compounds. The representative ICAs are SOD and CAT mimics. The low-valence metal ions in those enzymes reduce O_2_^•−^, and the high-valent metal ion formed in that way oxidizes a second molecule of the toxin. DCAs require the help of other cofactors to complete their full catalytic cycle. GPx and Prx mimics are representative DCAs that require GSH and Trx, respectively, to reduce H_2_O_2_ to H_2_O. Catalytic antioxidants, specialized classes of organometallic complexes, are mimics of SOD, CAT, or GPx that can detoxify a broad range of ROS [12,89].

### 3.1. Catalytic Antioxidants as SOD and CAT Mimics

SODs are ubiquitous metalloproteins that act as the first line of defense enzymes against ROS via the dismutation of O_2_^•−^ to H_2_O_2_ and molecular oxygen. Since heme is a naturally discovered native metalloporphyrin, Fe porphyrins, FeTM-4-PyP^5+^, were the first compounds proposed as SOD mimics in the late 1970s [90]. However, manganese (Mn) complexes remain the most stable and potential SOD mimics [91,92]. Currently, the main types of Mn-SOD mimetics are Mn cyclic polyamines, Mn and Fe porphyrin, Mn salen, and non-metal compounds such as nitrones and nitroxides [93]. Of the known synthetic compounds, nitrones and nitroxides cannot catalytically scavenge O_2_^•−^, but they can react with ONOO^−^.

#### 3.1.1. Macrocyclics 

Macrocyclics contain an Mn atom coordinated to five nitrogen ligands. Riley et al. designed Mn(II) cyclic polyamines, which is an optimized SOD mimetic (M40403, M series by Metaphore Pharmaceuticals) [94]. M40403 is a stable low molecular, Mn-containing, non-peptidic molecule that has the similar function and efficacy of native SOD enzymes [94]. The pentavalent coordination allows the Mn to participate in only single-electron transfers, which makes the compound specific for O_2_^•−^ scavenging because H_2_O_2_ or ONOO^−^ scavenging requires two-electron transfers [12,95,96]. In the kidney, only one study has demonstrated that M40403 protected gentamicin-induced AKI by suppressing nitrotyrosine formation and poly(ADP-ribose) synthetase activation [97]. 

#### 3.1.2. Mn porphyrins 

Metalloporphyrins are cell-permeable, redox-active, catalytic antioxidants that act as SOD mimetics, and have been demonstrated as potent catalysts of numerous redox reactions. Most metalloporphyrins have either a Fe or Mn moiety coordinated in four nitrogen axial ligands. Mn porphyrins (MnPs), the most potent Mn-SOD mimics, have been optimized to accumulate in the mitochondria, where they similarly act at the Mn-SOD catalytic site [89,98]. *Meso*-substituted metalloporphyrin analogs have varying degrees of SOD activity, net charge, and pharmacodynamic characteristics [99,100]. Generally, metalloporphyrins with stronger SOD activity possess greater CAT activity, but the CAT activity of SOD mimetics is less than 1% that of native CAT [89].

The Mn moiety of the SOD mimetics functions in the dismutation reaction with O_2_^•−^ by alternately reducing and oxidizing, which changes its valence from Mn(III) to Mn(II), much like native SODs. The O_2_^•−^ dismutation process of MnPs consists of two steps in which the Mn center cycles between Mn(III) and Mn(II). In the first step, Mn(III) reduced by O_2_^•−^ to yield Mn(II) and O_2_, and this step is considered as the rate-limiting step. The second step is the oxidation of Mn(II) by O_2_^•−^ to yield H_2_O_2_ and regenerate the Mn(III) porphyrins. This catalytic cycle is obviously modulated by the redox potential of the metal site [92]. The antioxidant efficiency of MnPs in vivo depends on their bioavailability, i.e., tissue, cellular, and subcellular distribution, and the nature of N-pyridyl substituents, which can alter their charge, size, shape, and lipophilicity [98,101,102,103].

MnP-based SOD mimics have been designed to imitate the kinetics and thermodynamics of SOD enzymes during the catalysis of O_2_^•−^ dismutation [104]. Since Irwin Fridovich first developed MnPs as potential SOD mimics in 1994 [105], diverse MnPs have been synthesized as cellular redox modulators. The first MnP-based lead compound was the cationic porphyrin Mn(III) *meso*-tetrakis(N-methylpyridinium-2-yl)porphyrin (MnTM-2-PyP^5+^, AEOL10112), along with Mn(III) *meso*-tetrakis(N-methylpyridinium-4-yl)porphyrin (MnTM-4-PyP^5+^) and the anionic porphyrin Mn(III) tetrakis(4-benzoic acid)porphyrin (MnTBAP^3−^, AEOL10201) (AEOL series by Aeolus Pharmaceuticals). In a next step of drug development, the ethyl analogue, Mn(III) *meso*-tetrakis(N-ethylpyridinium-2-yl)porphyrin (MnTE-2-PyP^5+^, AEOL10113, BMX-010) was synthesized [106,107]. MnTE-2-PyP^5+^ has increased bulkiness relative to MnTM-2-PyP^5+^, which reduces its interactions with nucleic acids and thereby its toxicity. Therefore, MnTE-2-PyP^5+^ emerged as one of the potent synthetic SOD mimetics and an effective ONOO^−^ scavenger. Mn(III) *meso*-tetrakis(1,3-diethylimidazolium-2-yl)porphyrin (MnTDE-2-ImP^5+^, AEOL 10150) is structurally different from MnTE-2-PyP^5+^, which possesses imidazole side chain substitutions. MnTDE-2-ImP^5+^ has kinetics and thermodynamics similar to MnTE-2-PyP^5+^, but it is bulkier, so it has different bioavailability.

Later investigators demonstrated the relationship between MnP lipophilicity and its biological activity. More lipophilic SOD mimics are effective at lower concentrations, whereas less lipophilic compounds are highly effective only at concentrations above 10 μM [108]. Therefore, the next stages in the drug development of MnP were improvements in lipophilicity that produced Mn (III) *meso*-tetrakis(N-n-hexylpyridinium-2-yl)porphyrin (MnTnHex-2-PyP^5+^) and decreases in toxicity while maintaining lipophilicity, which produced Mn(III) *meso*-tetrakis(N-n-butoxyethyl-pyridinium-2yl)porphyrin (MnTnBuOE-2-PyP^5+^, BMX-001) [103,109]. MnTnHex-2-PyP^5+^ has received much attention because it is significantly more lipophilic than MnTE-2-PyP^5+^ while having the same catalytic activity to eliminate O_2_^•−^ and ONOO^−^ [110]. Due to potent lipophilicity, MnTnHex-2-PyP^5+^ is distributed at the highest levels to all organs and accumulates in mitochondria better than most of the other analogs; it also shows decreased toxicity because of its micellar character [111]. MnTnHex-PyP^5+^ has a better therapeutic option than MnTE-2-PyP^5+^ due to its high bioavailability. To date, several MnPs, including MnTE-2-PyP^5+^ and MnTnBuOE-2-PyP^5+^, are currently being tested in clinical trials for cancerous and non-cancerous conditions [112].

Several cationic MnPs have been investigated in various models of kidney injury. Previous research reported that MnTM-4-PyP^5+^ administration attenuated tubulointerstitial damage in I/R injury by inhibiting apoptosis and proinflammatory cytokines [113,114]. Park et al. demonstrated that long-term administration of MnTM-4-PyP^5+^ ameliorated renal fibrosis after ischemic AKI by decreasing the deposition of collagen and accelerating the normalization of primary cilia length [115,116]. The same researchers later demonstrated the renoprotective mechanism of MnTM-4-PyP^5+^ using UUO mice. They found that it decreased ROS and prevented the elongation of primary cilia by inhibiting phosphorylated ERK, p21, and exocyst complex members Sec8 and Sec10 [117]. Another MnP, MnTnHex-2-PyP^5+^, also protected against renal I/R injury by inducing the production of ATP synthase-β subunit [110]. Similarly, the administration of MnTE-2-PyP^5+^, MnTM-4-PyP^5+^, or MnTM-2-PyP^5+^ conferred protection against the harmful effects associated with sepsis-induced AKI [118,119] and against diabetic nephropathy [120,121].

MnTBAP^3−^ compounds were initially developed as stable and efficient anionic SOD mimics [122], but later neither SOD-like activity nor CAT-like activity of MnTBAP^3−^ has been elucidated [123]. MnTBAP^3−^ was not efficacious due to its poor kinetics and thermodynamics; negative charges repelled this compound from the negatively charged deprotonated protein cysteines. Therefore, Rebouças and colleagues suggested that pure MnTBAP^3−^ could not interact with protein cysteines and catalyze H_2_O_2_ dismutation in aqueous media [124]. They suggested that MnTBAP^3−^ has often been inappropriately described as a SOD- and CAT-mimic and that its therapeutic effects have been erroneously assigned to SOD-like activity [103,124]. In addition, pure MnTBAP^3−^ can partially reduce ONOO^−^, but only if it is administered at high concentrations [125].

Despite the controversy, several experimental studies have demonstrated the renoprotective effects of MnTBAP^3−^ in various models of kidney disease. Zahmatkesh et al. showed that administering MnTBAP^3−^ prior to ischemia prevented renal I/R injury without changing plasma NOx levels [126,127]. Therefore, they suggested that MnTBAP^3−^ is not a NO scavenger and that its action could be mediated by the inhibition of ONOO^−^ production. Similarly, MnTBAP^3−^ attenuated cisplatin-induced nephrotoxicity by enhancing HO-1 and reducing nitrative stress [128]. Other researchers reported that MnTBAP^3−^ reduced ROS production and mitochondrial dysfunction by inhibiting the NLR family pyrin domain containing 3 (NLRP3) inflammasome and subsequently releasing proinflammatory cytokines in animal models of albumin- and aldosterone-induced renal tubular injury [129,130]. MnTBAP^3−^ also prevented tubulointerstitial fibrosis and mitochondrial dysfunction by reducing the deposition of extracellular matrix components, including fibronectin, collagen I, and collagen III, in mice with 5/6 nephrectomy [131].

#### 3.1.3. Manganosalens

The Mn(III)-containing salen compounds, i.e., EUK series (EUK series by Eukarion), are Mn complexes with a semi-cyclic ligand salen [12,92]. They have the catalytic activity of SOD, CAT, and peroxidase, and their mechanism of action is similar to those of the metalloporphyrins [132]. The EUK compounds have been shown to scavenge O_2_^•−^ and H_2_O_2_, react with ONOO^−^, and maybe react with lipid peroxides [12,65,133]. Mn(III) salens have modest SOD-like activity, whereas Mn(II) cyclic polyamines and Mn(III) porphyrins possess high SOD-like activity [98]. The prototype salen Mn complex (EUK-8) and the improved CAT mimetics (EUK-134 and EUK-189) are effective in a wide range of disease models, including kidney disease [15,133].

In the kidney, several experiments using both EUK-8 and EUK-134 have been performed. EUK-134 prevented renal dysfunction and tubulointerstitial injury by reducing oxidative and nitrosative stress in renal I/R injury [134,135]. In renal proximal tubular cells, EUK-134 significantly improved cell viability and reduced paraquat-induced cell death by reducing the production of O_2_^•−^ and OH^•^ [136]. EUK-8 attenuated lipopolysaccharide (LPS)-induced renal injury and delayed hypotension caused by endotoxins [137], and EUK-134 also prevented the LPS-induced fall in renal blood flow, which was associated with a decrease in protein nitrotyrosinylation in the kidney [138]. In an in vitro model of CKD, endothelial cells exposed to serum from uremic patients decreased their expression of intercellular adhesion molecule (ICAM)-1 and increased the phosphorylation of p38 mitogen-activated protein kinase (p38MAPK)-NF-κB signaling, and EUK-118 and EUK-134 treatment significantly decreased both intracellular ROS and phosphorylated p38MAPK-NF-κB expression [139].

#### 3.1.4. Nitroxides

Nitroxides, including tempol and Mito-TEMPO, are another class of non-metal-based SOD mimetics. Tempol (4-hydroxy-2,2,6,6-tetramethylpiperidine-N-oxyl) is a redox-cycling, water-soluble nitroxide that exhibits SOD-like activity and O_2_^•−^ scavenging activity [140]. Tempol is among the most potent of the nitroxides in protecting cells and tissues against ROS, but it does not sustain significant metabolism for more than a few hours due to a rapid exchange between the nitroxide, hydroxylamine, and oxammonium cation species [141]. To date, the renoprotective effects of tempol have been demonstrated in numerous experimental studies of diverse kidney diseases, especially hypertensive and diabetic kidney disease. Pretreatment with tempol attenuated renal dysfunction and decreased ROS in renal I/R injury and LPS-induced AKI models [142,143,144]. The decrease of SOD activity in diabetic nephropathy was widely known through previous experiments, and tempol treatment in diabetic nephropathy restored renal function and the activity of antioxidant enzymes, including SOD and GPx [42,145,146,147]. These effects were attributed to improved endothelial function [145], reduced renal vascular resistance associated with HO-1 expression [146], and the upregulation of transient receptor potential cation channel subfamily C member 6 (TRPC6) expression [147]. Consistent with the results from diabetic nephropathy, obese, diabetic, hypertensive ZSF_1_ rats treated with tempol showed increased SOD activity and significantly reduced lipid peroxidation and peroxidase activity in the kidney [148].

Since hypertension and renal vasoconstriction depend on O_2_^•−^, the biological effects of tempol on endothelial function have been studied extensively in various animal models of hypertension. Tempol treatment decreased the mean arterial pressure by decreasing the renal sympathetic nerve response [149], increasing plasma renin activity [150], and increasing medullary blood flow and sodium excretion [151] in hypertensive animal models, such as spontaneously hypertensive rats and fructose-hypertensive rats. Nishiyama et al. also demonstrated that tempol protects against glomerular injury by inhibiting MAPK and NOX signaling in a salt-dependent model of hypertension [152]. Another chronic renal hypoxia model using the two-kidney, one-clip hypertension technique decreased SOD1 expression, especially in the tubulointerstitial area, which was associated with increased TNF-α. Tempol treatment ameliorated tubulointerstitial injury and reduced macrophage infiltration in the renovascular hypertensive model [153]. Chronic Ang II infusion was also accompanied by extensive renal fibrosis, represented as upregulated NOX and suppressed SOD. Cotreatment with an NADPH inhibitor and tempol inhibited TGF-β1 expression and the related fibrogenic responses in the chronic Ang II infusion model of hypertensive kidney disease [154]. Consistent with chronic Ang II infusion, mice who underwent 5/6 nephrectomy downregulated SOD1 and SOD2, upregulated NOX, and increased atrial pressure and nitrotyrosine, and tempol treatment ameliorated the hypertension and increased the level of urinary NO metabolites [155]. 

Mito-TEMPO, a mitochondrial-targeted SOD mimetic, is a nitroxide linked to the triphenyl phosphonium cation, which promotes 1,000-fold accumulation into the mitochondrial matrix [156,157]. Mito-TEMPO restored renal mitochondrial function and attenuated sepsis-induced AKI by decreasing mitochondrial oxidative stress and increasing Mn-SOD activity [158]. Mito-TEMPO also prevented aldosterone-induced renal tubular injury by restoring mitochondrial function and suppressing the activation of the NLRP3 inflammasome and apoptosis [159]. In addition, mitochondrial dysfunction, inflammatory cytokine levels, oxidative stress, and endoplasmic reticulum (ER) stress were involved in 5/6 nephrectomy-induced renal fibrosis, and Mito-TEMPO attenuated tubulointerstitial fibrosis by ameliorating renal inflammation, mitochondrial dysfunction, and ER stress [160]. Furthermore, indoxyl sulfate treatment in the 5/6 nephrectomy model augmented renal fibrosis and decreased renal function by activating the NOX and RhoA/Rho-associated kinase (ROCK) pathway; Mito-TEMPO or tempol decreased NOX and increased SOD1 and SOD2 in the thoracic aorta of indoxyl sulfate-treated 5/6 nephrectomy model [161].

### 3.2. Catalytic Antioxidants as GPx Mimics 

#### 3.2.1. Ebselen

Organoselenium compounds exhibit potent antioxidant activity mediated by GPx mimetic properties. Ebselen (2-phenyl-1,2-benzisoselenazol-3(2H)-one or PZ51), the first Se-based GPx mimic, is one of the best studied GPx mimics [65]. Ebselen can metabolize peroxides using GSH or directly reduce thioredoxin reductase [11]. Ebselen also reduces H_2_O_2_ and lipid peroxides and scavenges ONOO^−^ without affecting endogenous NO^•^ [162]. Ebselen was less toxic than other treatments tested because of its stable isoselenazole moiety, and it proved to be an effective treatment for experimental models of diverse kidney diseases. The beneficial effects of ebselen were mainly investigated in cisplatin-induced AKI models [163,164,165,166]. Baldew et al. first demonstrated that pretreatment with ebselen prevented cisplatin-induced renal injury and that the protective effect of ebselen was dose-dependent [163]. Other researchers reported that ebselen treatment enhanced the activities of antioxidant enzymes such as SOD, CAT, and GPx without changing the cisplatin concentrations in cisplatin-induced AKI [165,166]. Ebselen treatment also prevented AKI from other causes, including gentamycin, I/R injury, and radiocontrast [167,168,169].

Ebselen also proved to be an effective treatment for reducing oxidative stress of the kidney in various models of diabetic nephropathy, including Zucker diabetic fat rats [170], diabetic ApoE^−/−^ GPx^−/−^ mice [76,171], and STZ-induced diabetic mice [172]. Ebselen treatment prevented decreases in capillary density and angiogenic competence related to vascular endothelial growth factor (VEGF) expression and restored acetylcholine-induced vasorelaxation in Zucker diabetic fat rats [170]. A recent study demonstrated that ebselen improved endothelial dysfunction by increasing endothelial GSH levels and reducing p38MAPK and NF-κB activation in uremic sera-exposed endothelial cells [139]. Ebselen was also shown to improve diabetes-associated atherosclerosis and renal injury by reducing oxidative stress and proatherogenic markers such as VEGF, connective tissue growth factor (CTGF), vascular cell adhesion molecule-1 (VCAM-1), and monocyte chemoattractant protein-1 (MCP-1) in diabetic ApoE^−/−^ GPx^−/−^ mice [76]. However, ebselen attenuated albuminuria and renal dysfunction in diabetic mice only with early intervention; it did not alter albuminuria and glomerulosclerosis with late intervention [172].

#### 3.2.2. Diphenyl Diselenide

Diphenyl diselenide (PhSe2) is another organoselenium compound that has been reported to catalytically scavenge peroxides, with higher GPx-like activity than ebselen [173]. However, the diphenyl diselenides are electrophilic agents with cytotoxic, genotoxic, and mutagenic effects [174]. Diphenyl diselenide prevented the inhibition of δ-aminolevulinate dehydratase (δ-ALA-D), CAT and GPx activity, and enhanced ascorbic acid levels in glycerol-induced AKI [175]. In contrast, diphenyl diselenide in mercuric chloride nephropathy potentiated renal damage and oxidative stress, compared with mercuric chloride alone [176]. Diphenyl diselenide also attenuated STZ-induced toxicity by increasing platelet nucleoside triphosphate diphosphohydrolases (NTPDases) and 5′-nucleotidase, inhibitors of platelet aggregation, without increasing δ-ALA-D and Na^+^K^+^-ATPase [177]. A recent study reported that diphenyl diselenide was as effective as ebselen in treating cisplatin-induced AKI, and its mechanism was reducing oxidative stress by activating δ-ALA-D and Na^+^/K^+^-ATPase and upregulating the Nrf2/Keap-1/HO-1 pathway [178].

## 4. Conclusions

In this review, we discussed the role of antioxidant enzymes and the emerging evidence for the renoprotective effects of catalytic antioxidants in kidney disease (Table 1). Catalytic antioxidants, especially mimics of specific redox enzymes such as SOD, CAT, and GPx, have been demonstrated to have therapeutic advantages in various experimental models of kidney disease. Despite the protection against ROS shown by these compounds in in vitro and in vivo oxidative stress models, their practical application in kidney disease remains highly challenging. Therefore, further clinical trials are needed to assess the efficacy and toxicity of catalytic antioxidants in the human body and confirm their clinical applications in kidney disease. We expect this review to be helpful to researchers developing catalytic antioxidants applicable to various kidney diseases.

## Figures and Tables

**Table 1:** Summary of in vitro and in vivo trials of catalytic antioxidants in kidney diseases.

Groups	Structures	Compounds	Diseases	Models	Species or Cells	Ref.
SOD mimics: metal-based	Macrocyclics	M40403	AKI	Gentamycin	Rats	[97]
	Mn Porphyrins	AEOL10112 (MnTM-2-PyP^5+^)	DKD	Streptozotocin	Rats	[120,121]
		AEOL10113 (MnTE-2-PyP^5+^)	AKI	Lipopolysaccharide	Mice	[119]
		MnTM-4-PyP^5+^	AKI	I/R injury	Rats, Mice	[113,114,115,116]
				Cecal ligation and puncture	Mice	[118]
			CKD	Unilateral nephrectomy	Mice	[117]
		MnTnHex-2-PyP^5+^	AKI	I/R injury	Rats	[110]
Non-SOD mimics: metal-based	Mn Porphyrins	AEOL10201 (MnTBAP^3-^)	AKI	I/R injury	Rats	[126,127]
				Cisplatin	Mice	[128]
			CKD	Albumin	Mice	[129]
				Aldosterone	Mice/HK-2 cells	[130]
				5/6 nephrectomy	Mice/mPT cells	[131]
SOD/CAT mimics: metal-based	Mn Salens	EUK-134	AKI	I/R injury	Rats	[134,135]
				Paraquat	NRK-52E cells	[136]
				Lipopolysaccharide	Pigs	[138]
		EUK-8	AKI	Lipopolysaccharide	Rats	[137]
		EUK-118, EUK-134	CKD	Uremic media	Endothelial cells	[139]
SOD mimics: Non-metal-based	Nitroxides	Tempol	AKI	Paraquat	NRK-52E cells	[136]
				I/R injury	Rats	[142,143]
				Lipopolysaccharide	Rats	[144]
			DKD	Streptozotocin	Rats	[145,146,147]
				KK/Ta-Akita mice	Mice	[42]
			Obesity	ZSF_1_ rats	Rats	[148]
			HTN	Spontaneously hypertensive rats	Rats	[149,150]
				Dahl salt-resistant rats	Rats	[152]
				Unilateral renal artery stenosis	Rats	[153]
				Angiotensin II	Rats	[154]
				Fructose	Rats	[151]
			CKD	5/6 nephrectomy	Rats	[155]
				5/6 nephrectomy + IS	Rats	[161]
		Mito-TEMPO	AKI	Cecal ligation and puncture	Mice	[158]
			CKD	5/6 nephrectomy + IS	Rats	[161]
				5/6 nephrectomy	Mice	[160]
				Aldosterone	Mice	[159]
GPx mimics	Organoselenium	Ebselen	AKI	Cisplatin	Rats, Mice, LLC-PK1 cells	[163,164,165,166]
				Gentamycin	Rats	[167]
				I/R injury	Rats	[168]
				Radiocontrast	Rats	[169]
				Gentamycin	Rats	[167]
			CKD	Uremic media	Endothelial cells	[139]
			DKD	Akita mice	Mice	[172]
				ApoE/GPx1 dKO	Mice	[76,171]
				Zucker diabetic fat	Rats	[170]
		Diphenyl diselenide	AKI	Cisplatin	Rats	[178]
				Mercuric chloride	Mice	[176]
				Glycerol	Rats	[175]
			DKD	Streptozotocin	Rats	[177]

**Abbreviations:** AKI; acute kidney injury, ApoE; Apolipoprotein E, CAT; catalase, CKD; chronic kidney disease, DKD; diabetic kidney disease, dKO; double knock out, GPx; glutathione peroxidase, HK-2; human kidney-2, HTN; hypertension, I/R; ischemia/reperfusion, IS; indoxyl sulfate, LLC-PK1; Epithelial-like pig kidney1, Mn; manganese, mPT; mouse proximal tubule, NRK-52E; normal rat kidney-52E, SOD; superoxide dismutase. AEOL series, EUK series, and M series are currently being developed by Aeolus Pharmaceuticals, Eukarion, and Metaphore Pharmaceuticals, respectively.

## Data Availability

No new data were created or analyzed in this study. Data sharing is not applicable to this article.
